# Investigation of the association between the *TCF7L2* rs7903146 (C/T) gene polymorphism and obesity in a Cameroonian population: a pilot study

**DOI:** 10.1186/s41043-017-0087-z

**Published:** 2017-04-18

**Authors:** Aurelie Nguimmo-Metsadjio, Barbara Atogho-Tiedeu, Jean Jacques N. Noubiap, Marie-Solange Evehe, Rosine Djokam-Dadjeu, Olivier Sontsa Donfack, Dieudonne Nanfa, Edith Pascale M. Mato, Elvis Ndonwi Ngwa, Magellan Guewo-Fokeng, Priscille Pokam-Fosso, Wilfred F. Mbacham, Jean Claude Mbanya, Eugene Sobngwi

**Affiliations:** 1 Department of Biochemistry, Faculty of Science, University of Yaoundé I, Yaoundé, Cameroon; 2 Laboratory for Molecular Medicine and Metabolism, Biotechnology Center, University of Yaoundé I, Yaoundé, Cameroon; 3 Department of Medicine, Groote Schuur Hospital and University of Cape Town, Cape Town, South Africa; 4 Medical Diagnostic Center, Yaoundé, Cameroon; 5 Laboratory for Public Health Research Biotechnologies, Biotechnology Center, University of Yaoundé I, Yaoundé, Cameroon; 6 Department of Internal Medicine and Specialties, Faculty of Medicine and Biomedical Sciences, University of Yaoundé I, Yaoundé, Cameroon; 7 National Obesity Center, Yaoundé Central Hospital, Yaoundé, Cameroon

**Keywords:** Obesity, Genetic association, TCF7L2, rs7903146 (C/T) polymorphism, Sub-Saharan Africa, Cameroon

## Abstract

**Objective:**

This study aimed at investigating the association between the rs7903146 (C/T) polymorphism of the *TCF7L2* gene with obesity in a Cameroonian population.

**Method:**

This was a case-control pilot study including 61 obese and 61 non-obese Cameroonian adults. Anthropometric indices of obesity, blood pressure, fasting blood glucose, and blood lipids were measured. The rs7903146 (C/T) polymorphism of the *TCF7L2* gene was genotyped using polymerase chain reaction-restriction fragment length polymorphism (PCR-RFLP), and genotypes were correlated with clinical and biological parameters.

**Results:**

The T allele was predominant in the study population with a frequency of 93%. No statistically significant difference was however observed between the genotypic (*p* = 0.50) and allelic frequencies (*p* = 0.58) of obese and non-obese subjects. Comparison of clinical and biochemical parameters of C allele carriers (CX = CC + CT) with those of TT genotype showed that there was no significant difference between the lipid profile of these two groups.

**Conclusion:**

The rs7903146 (C/T) polymorphism of the *TCF7L2* gene might not be associated with obesity in the Cameroonian population.

## Introduction

Obesity is a major public health problem worldwide, reporting a high prevalence (2.1 billion individuals affected) in 2013 and a high mortality (3.4 related deaths) in 2010 [1]. With an estimated prevalence of overweight or obesity among adults aged ≥15 years which increased from 22.5% in 2002 to 26% in 2006, Cameroon is also affected by the epidemic [2, 3].

The development of this disease is influenced by a variety of external factors that may be environmental, psychological, and/or genetic [4]. There is overwhelming evidence that weight is partially genetically determined with an estimated heritability varying between 6 and 85% [5]. Nevertheless, genetic pathways that contribute to obesity are yet to be elucidated.

In 2006, researchers of the deCODE project based in Iceland identified *TCF7L2* gene polymorphisms in a binding region of chromosome 10 that were strongly associated with the risk of type 2 diabetes [6]. This association has been replicated in several ethnic groups, with an allele relative risk of 1.4. This is the strongest association observed among the genes generally associated with type 2 diabetes [7]. The protein derived from the *TCF7L2* (transcription factor 7-like 2) gene, also known as the TCF4, is an important transcription factor in the Wnt signaling pathway [8], which was initially characterized in colon cancer and in the embryonic development of Drosophila, Xenopus, and other organisms [9]. It was shown that in adipocytes, Wnt signaling via *TCF7L2* downregulates adipogenesis [10]. In a study among Caucasians, the effect of TCF7L2 on T2DM was modulated by obesity [11]. Furthermore, a recent study has shown that changes in weight may be influenced by the TCF7L2 rs7903146 variant [12]. Likewise, this genetic variant was associated with obesity in a Saudi population [13].

To the best of our knowledge, there is no published data on the role of *TCF7L2* in obesity among sub-Saharan African populations. In a recent study, we showed that the TCF7L2 rs7903146 (C/T) gene polymorphism may be associated to type 2 diabetes in Cameroonians [14]. The current pilot study aimed at investigating the association between this genetic variant and obesity in a Cameroonian population.

## Methods

### Ethical considerations

Ethical clearance was obtained from the National Ethics Review Board of the Cameroon Ministry of Public Health. The study was conducted in accordance with the Helsinki Declaration. All participants provided written informed consent.

### Study population

We conducted a case-control study involving subjects of Cameroonian origin, aged 18 years old and above. Obese patients were recruited from the outpatient clinic of the National Obesity Center of the Yaoundé Central Hospital, and non-obese controls from the general population after a radio announcement were invited to participate in the study. We excluded known diabetic patients, those who had a fasting blood glucose level ≥1.26 g/l and pregnant women. We finally included 61 obese subjects (body mass index (BMI) ≥30 kg/m^2^) and 61 normal weight controls (BMI 18.5–24.9 kg/m^2^).

### Data collection

#### Clinical data

After a radio announcement asking for volunteers, male and female individuals presented themselves at the National Obesity Center where information on the study and recruitment criteria (BMI ≥30 kg/m^2^ and 18.5 ≤ BMI <25, age ≥18 years old) was given. The following day, participants who met the inclusion criteria and desired to participate in the study presented themselves at approximately 8 a.m. at the National Obesity Center after an overnight fast of at least 8 h. After responding to a questionnaire on lifestyle (diet, physical activity), we measured the height, waist, and hip circumference to the nearest 0.5 cm and weight in light clothes to the nearest 0.1 kg, and we then calculated the BMI as weight in kg/height^2^ in m^2^ and the waist-to-hip ratio (W/H) which is a measure of central obesity. We measured the blood pressure using a standardized protocol with the participant in a seated position, and after at least 10 min of rest, with a validated automated blood pressure measuring device (OMRON HEM-757). Fasting blood glucose of each participant was measured from capillary blood using the One Touch Ultra® 2 (LifeScan, Inc., Milpitas, CA 95035, USA) device.

#### Biochemical assays

Blood was collected in one EDTA tube and one dry tube by venipuncture from each fasting individual. Serum was obtained from the blood in the dry tubes after centrifugation and stored at 4 °C for biochemical analyses. Total cholesterol, high-density lipoprotein (HDL) cholesterol, low-density lipoprotein (LDL) cholesterol, and triglycerides were measured by enzymatic colorimetric method. Total cholesterol was determined by a method derived from that described by Allain et al. [15] and triglycerides by a method derived from that of Buccolo and Davids [16]; HDL cholesterol levels were measured in the supernatant by the method of Burstein et al. [17] after precipitation of chylomicrons, LDL cholesterol, and VLDL, with phosphor-tungstic acid and magnesium chloride. LDL cholesterol was calculated using Friedwald’s formula [18].

#### DNA extraction and genotyping

Blood samples in EDTA tubes were used for DNA extraction and genotyping. Genomic DNA was extracted from blood leukocytes of the participants by the Chelex method [19]. The rs7903146 (C/T) polymorphism of the *TCF7L2* gene was analyzed using the polymerase chain reaction-restriction fragment length polymorphism (PCR-RFLP) method with the following primers: forward 5′-AAG AGA AGA TTC TTC TTT AAA TGG TG-3′ and reverse 3′-CTC CAT CAG GCA AAA TTA TAC ATT A-5′ (SIGMA-ALDRICH, St. Louis, MO, USA). A final reaction volume of 15 μL for the polymerase chain reaction (PCR) was constituted, which contained 100 ng of genomic DNA, 5 pmol of each primer, PCR buffer with 1 mmol/L of MgCl_2_, 100 μmol/L of each deoxynucleotide triphosphate (dNTP), 0.5 U of Hot Start *Taq* DNA polymerase (QIAGEN), and 7.8 μl of nuclease-free water. The PCR was carried out on a BIOMETRA T3 thermal cycler under the following conditions: 95 °C for 2 min followed by 34 cycles of 95 °C for 30 s, 58 °C for 30 s, 72 °C for 30 s, and a final extension of 72 °C for 5 min. The amplicons were analyzed by agarose gel electrophoresis using a 2% agarose gel and positive amplicons digested with *Helicobacter pylori* CH4 III (*Hpy*-CH4III) restriction enzyme at 37 °C overnight. The resulting products were separated by electrophoresis on a 3% agarose gel and visualized under a UV transilluminator.

### Statistical analyses

Statistical analysis was performed using STATA 11.0. The Shapiro-Wilk test was used to test the normality of the data. Categorical variables were expressed as frequencies and percentages and continuous variables as medians and interquartile ranges (IQR). The chi-square goodness-of-fit test was used to test the Hardy-Weinberg equilibrium of the study population. Allele and genotype frequencies were compared using Fisher’s exact test. Differences in clinical and biological parameters were compared between groups by non-parametric Mann-Whitney test or Kruskal-Wallis followed by a post-comparison with the Dunn-Sidak test. The significance level was set at 5% and 0.05/*k* for multiple comparison tests (*k* being the number of pairs of comparisons).

## Results

### Clinical and biochemical characteristics of the study population

Table 1 shows the clinical and biochemical characteristics of the study population which consisted of 96 women and 26 men. A higher proportion of cases than controls were women. Similarly, age was significantly higher (*p* < 0.0001) in obese (median 45 years, IQR 38.5–51.5) compared to that in non-obese (median 30 years, IQR 24.0–42.5) participants. Comparison of clinical and biochemical parameters between obese and non-obese volunteers showed that the systolic blood pressure (SBP) and diastolic blood pressure (DBP) were significantly higher (*p* < 0.0001) in the obese group, as well as fasting plasma glucose (*p* = 0.015).

**Table 1:** Clinical and biochemical features

Characteristic	Non-obese	Obese	*p* value
Sex (F/M)	40/21	56/5	<0.0001
Age (year)	30 (24–42.50)	45 (38.50–51.50)	<0.0001
W/H ratio	0.80 (0.76–0.85)	0.85 (0.82–0.91)	<0.0001
SBP (mmHg)	114 (104–125)	126 (116–141)	<0.0001
DBP (mmHg)	68 (63–79)	79 (72–88)	<0.0001
BMI (kg/m^2^)	22.23 (21.01–23.92)	32.73 (30.94–36.52)	<0.0001
% fat	25.42 (21.59–29.19)	43.44 (40.52–47.96)	<0.0001
FBG (mg/dl)	91 (83–98)	97 (87–104)	0.015
TC (mg/dl)	175.5 (163.2–190.5)	183.6 (171–205.5)	0.080
HDL-C (mg/dl)	47.18 (44.04–50.24)	47.02 (43.89–50.63)	0.76
LDL-C (mg/dl)	101.8 (91.82– 116.1)	110.5 (95.78–134.3)	0.14
TG (mg/dl)	128(120.9–141.6)	131.4 (122–143.3)	0.33

The results are expressed as median (interquartile range)

*W/H ratio* ratio of waist-to-hip circumference, *SBP* systolic blood pressure, *DBP* diastolic blood pressure, *BMI* body mass index, *FBG* fasting blood glucose, *TC* total cholesterol, *HDL-C* HDL cholesterol, *LDL-C* LDL cholesterol, *TG* triglycerides

### Association between rs7903146 (C/T) polymorphism of *TCF7L2* gene and obesity

**Fig. 1:**
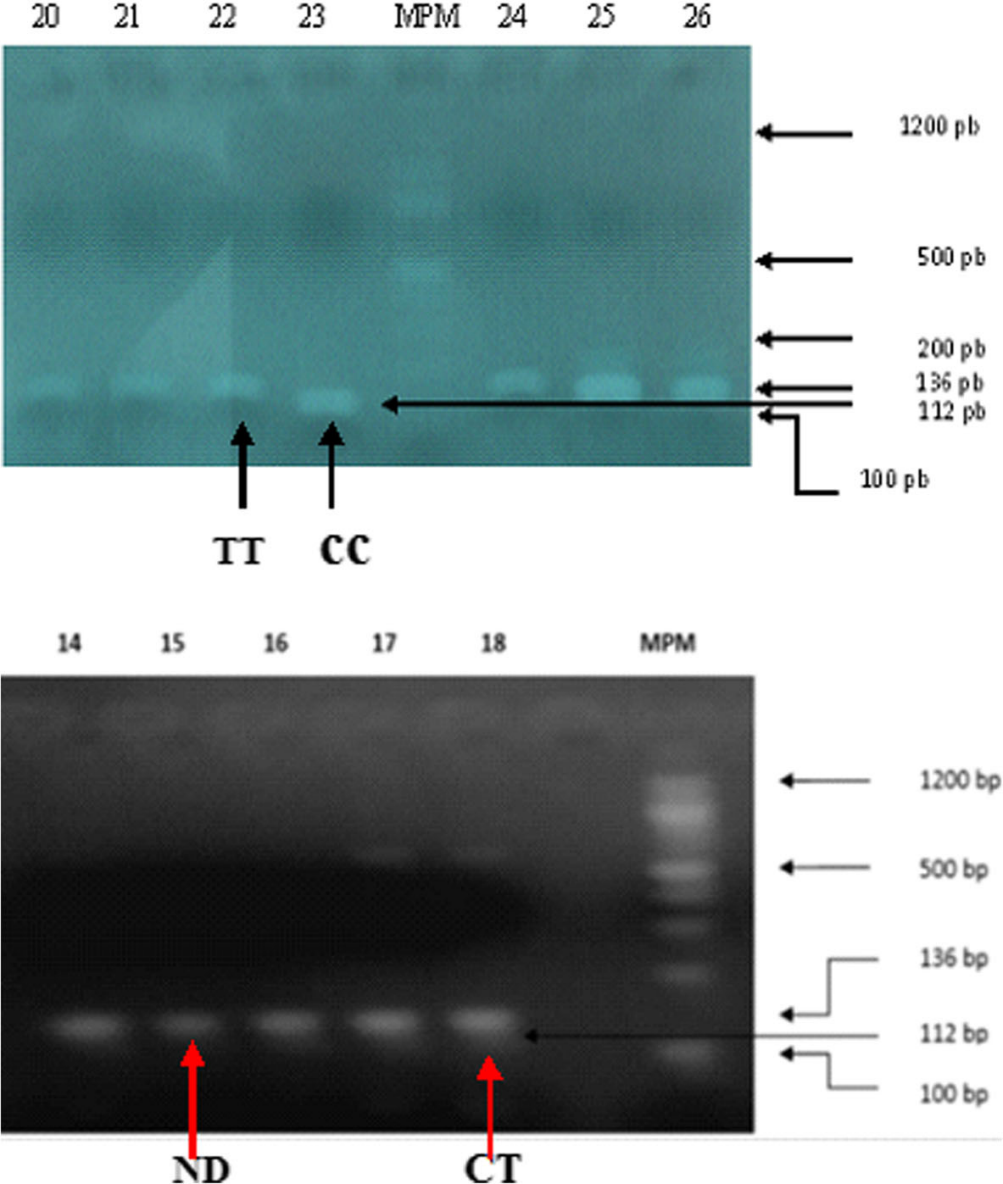
Digestion profile of TCF7L2 rs7903146 (C/T) amplicons with *Hpy*-CH4III. The expected product sizes are 136 bp (mutant homozygote TT), 112 bp (wild homozygote), and 136 and 112 bp (heterozygote) respectively. *MPM* 100 bp molecular weight marker; fragments smaller than 100 bp were not visualized. *ND* non-digested amplicon. Participant numbers: 14, 15, 16, 17, 18, 20, 21, 22, 23, 24, 25, 26

The study population was in Hardy-Weinberg equilibrium. After PCR amplification and digestion with *Hpy*-CH4III, two bands of 112 bp characteristic of the CC genotype, 90 bands of 136 bp for the TT genotype, and 10 bands of 112 and 136 bp for the CT genotype were visualized on a 3% agarose gel (Fig. 1). The CT genotype is blurred because of the low quality of the image. Table 2 shows the allele and genotype frequencies of the rs7903146 (C/T) polymorphism of the *TCF7L2* gene in the study population. No statistically significant difference was observed between the genotypic (*p* = 0.50) and allelic frequencies (*p* = 0.58) of obese and non-obese controls.

**Table 2:** Allele and genotype frequencies of the rs7903146 (C/T) polymorphism in the study population

rs7903146 (C/T)	Non-obese	Obese	*p* value
Alleles			
T	102 (94.4)	88 (91.66)	
C	6 (5.5)	8 (8.33)	0.58
Total (2*n*)	108	96	
Genotypes			
C/C	1 (1.85)	1 (2.08)	
C/T	4 (7.4)	6 (12.5)	
T/T	49 (90.7)	41 (85.41)	0.50
Total (*n*)	54	48	

The results are given as *n* (%)

### Stratification of clinical and biochemical parameters in relation to genotype

Table 3 shows the comparison between clinical and biochemical parameters of the individuals carrying the C allele compared to those with the TT genotype. According to this table, there was no difference in the clinical parameters between carriers of the C allele (CX = CC + CT) and those with genotype TT.

**Table 3:** Stratification of clinical and biological parameters among genotypes

Characteristic	CX (CC + CT) *n* = 12	TT *n* = 90	*p* value
W/H ratio	0.84 (0.81–0.87)	0.83 (0.78–0.89)	0.65
SBP (mmHg)	123 (116.5–132.5)	120 (107–135)	0.42
DBP (mmHg)	75.5 (69–81.5)	75.5 (67–85)	0.89
BMI (kg/m^2^)	30.1 (23.7–31)	24.4 (21.8–32.8)	0.66
FBG	95.5 (88–107)	92.5 (85–102)	0.29
TC (mg/dL)	195.5 (189.5–222.1)	179.8 (164.7–198.4)	0.02
TG (mg/dL)	135.2 (121.6–158.4)	130.1 (121.1–142.5)	0.38
HDL-C (mg/dL)	44 (42–50.8)	47 (44.7–50.2)	0.28
LDL-C (mg/dL)	122.9 (118.1–148.3)	103.5 (94.6–118.4)	0.01

The results are expressed in median (interquartile range)

*W/H ratio* ratio of waist to hip circumference, *SBP* systolic blood pressure, *DBP* diastolic blood pressure, *BMI* body mass index, *FBG* fasting blood glucose, *TC* total cholesterol, *HDL-C* HDL cholesterol, *LDL-C* LDL cholesterol, *TG* triglycerides

## Discussion

Despite the growing burden of obesity in Africa and the important contribution of genetic factors to the disease [1, 4, 5], data on the genetic variants in African populations are very scarce. Our study provides first-time insight into the role of the *TCF7L2* rs7903146 (C/T) gene polymorphism in obesity among Cameroonians. This genetic variant may not be associated with obesity in our study population.

TCF7L2 is a transcription factor involved in the Wnt signaling pathway which is a pathway involved in adipogenesis [10]. In vitro inhibition of the transcription factor has been associated with stimulation of adipogenesis [10]. *TCF7L2* is one of the genes that have been identified as possible determinants of obesity. Indeed, it was shown in a Caucasian population that the effect of TCF7L2 on T2DM is modulated by obesity. Precisely, data suggested that the rs7903146 T allele may be possibly functional and associated with a nominal decrease in TCF7L2 expression in adipose tissue of individuals under calorie restriction [11]. Klunder et al. showed a protective association between rs12255372 polymorphism and obesity in Mexican children, while a deleterious effect of this variant was observed in diabetic adults [20]. Furthermore, a recent study showed that changes in weight may be influenced by the TCF7L2 rs7903146 variant [12]. Likewise, this genetic variant was associated with obesity in a Saudi population [13]. However, our study showed no association between the TCF7L2 rs7903146 gene polymorphism and obesity. These results are similar to those obtained by Cauchi et al. who found no genetic association between the TCF7L2 rs7903146 gene polymorphism and obesity in European populations [11]. Absence of association with obesity was also demonstrated for other polymorphisms of TCF7L2 including rs10885406 in US populations [21].

The absence of association between TCF7L2 rs7903146 and obesity in our population may be explained by the small size of our study population. Unlike other ethnic groups, the T allele was predominant in our study population with a frequency of 93% [22]. Further studies with higher sample size would increase the probability of obtaining the C allele and would therefore be more powerful to verify our findings.

Moreover, the relatively small sample size of Cameroonian males was also a limitation to this study. This imbalance was due to the fact that participants were recruited consecutively following a radio announcement, and women were more prone to participate in the study. This imbalance was even more pronounced in the obese group because of the high prevalence of obesity among Cameroonian women [23]. According to a study carried out in 2003 by Pasquet and collaborators, one woman in five was obese, whereas only 5% of men were obese among Cameroonian urban adults [24].

Our study also showed that obesity is associated with an increase in waist-to-hip ratio (WHR). WHR has been suggested to be a superior predictor of cardiovascular disease (CVD) risk. It has been shown that a 0.01 increase in WHR is associated with a 5% increase in risk of future CVD [25]. Therefore, obese participants may be more prone to CVD than controls although their WHR values are within normal range.

## Conclusion

Our study shows that the rs7903146 (C/T) polymorphism might not be associated with obesity in the Cameroonian population. Our findings, which may be confirmed by further studies with larger sample size, specifically warrant investigation of the association between the TCF7L2 gene and blood lipids in various ethnic populations.

## Abbreviations

DNA: Deoxyribonucleic acid
PCR: Polymerase chain reaction
*TCF7L2*: Transcription factor 7-like 2
