# Erratum to: Investigation of the association between the *TCF7L2* rs7903146 (C/T) gene polymorphism and obesity in a Cameroonian population: a pilot study

**DOI:** 10.1186/s41043-017-0094-0

**Published:** 2017-05-16

**Authors:** Aurelie Nguimmo-Metsadjio, Barbara Atogho-Tiedeu, Jean Jacques Noubiap, Marie-Solange Evehe, Rosine Djokam-Dadjeu, Olivier Sontsa Donfack, Dieudonne Nanfa, Edith Pascale M. Mato, Elvis Ndonwi Ngwa, Magellan Guewo-Fokeng, Priscille Pokam-Fosso, Wilfred F. Mbacham, Jean Claude Mbanya, Eugene Sobngwi

**Affiliations:** 1 Department of Biochemistry, Faculty of Science, University of Yaoundé I, Yaoundé, Cameroon; 2 Laboratory for Molecular Medicine and Metabolism, Biotechnology Center, University of Yaoundé I, Yaoundé, Cameroon; 3 Department of Medicine, Groote Schuur Hospital and University of Cape Town, Cape Town, South Africa; 4 Medical Diagnostic Center, Yaoundé, Cameroon; 5 Laboratory for Public Health Research Biotechnologies, Biotechnology Center, University of Yaoundé I, Yaoundé, Cameroon; 6 Department of Internal Medicine and Specialties, Faculty of Medicine and Biomedical Sciences, University of Yaoundé I, Yaoundé, Cameroon; 7 National Obesity Center, Yaoundé Central Hospital, Yaoundé, Cameroon

## Erratum

Upon publication of the original article [1], it was noticed that the author’s name “Jean Jacques Noubiap” was incorrectly given as “Jean Jacques N. Noubiap”. This has now been acknowledged and corrected in this erratum.
